# Characterization of Polyester Coatings Intended for Food Contact by Different Analytical Techniques and Migration Testing by LC-MS^n^

**DOI:** 10.3390/polym14030487

**Published:** 2022-01-26

**Authors:** Antía Lestido-Cardama, Patricia Vázquez-Loureiro, Raquel Sendón, Juana Bustos, Mª Isabel Santillana, Perfecto Paseiro Losada, Ana Rodríguez Bernaldo de Quirós

**Affiliations:** 1 Department of Analytical Chemistry, Nutrition and Food Science, Faculty of Pharmacy, University of Santiago de Compostela, 15782 Santiago de Compostela, Spain; antia.lestido@usc.es (A.L.-C.); patriciavazquez.loureiro@usc.es (P.V.-L.); raquel.sendon@usc.es (R.S.); perfecto.paseiro@usc.es (P.P.L.); 2 National Food Center, Spanish Agency of Food Safety and Nutrition, E-28220 Majadahonda, Spain; JBustos@aesan.gob.es (J.B.); misabelsantillana@gmail.com (M.I.S.)

**Keywords:** polyester, can coating, GC-MS, MALDI TOF MS, LC-MS^n^

## Abstract

Polymeric coating formulations may contain different components such as cross-linking agents, resins, lubricants, and solvents, among others. If the reaction process or curing conditions are not applied in a proper way, these components may remain unreacted in the polymeric network and could be released and migrate into foods. In this study, several polyester coatings intended for food contact were investigated. Firstly, Fourier-transform infrared spectroscopy with an attenuated total reflectance (ATR-FTIR) spectrometer and confocal Raman microscopy were used to identify the type of coating. Then, different techniques, including gas chromatography coupled to mass spectrometry (GC-MS) and analysis by matrix-assisted laser desorption coupled to time-of-flight mass spectrometry (MALDI-TOF-MS), among others, were used to investigate the potential volatile and non-volatile migrants. Moreover, migration assays were carried out to evaluate the presence of monomers and to tentatively identify possible oligomers below 1000 Da. The analyses were performed by liquid chromatography coupled to ion trap mass spectrometry (LC-MS^n^). Using the information collected from each analytical technique, it was possible to elucidate some of the starting substances used in the formulation of the polyester coatings analyzed in this study. In migration tests, several polyester oligomers were tentatively identified for which there is not toxicological data available and, therefore, no migration limits established to date.

## 1. Introduction

Normally, metal cans intended to come into contact with food and beverages have an internal coating to protect the food from the metal and vice versa during pasteurization, sterilization, and long-time storage. Epoxy resins are the type of coating most widely used in metal substrates. However, can manufacturers have started to innovate and develop substitutes to replace food-contact materials based on epoxy resins as a consequence of reported toxic effects of their components and the limitations established by the regulatory authorities. Polyester-based coatings are currently used as a first-generation alternative to epoxy-based resins [1].

Polyester polymer coatings are produced from polycondensation reactions between carboxylic acid monomers, such as phthalic, isophthalic, or terephthalic acids, and poly-functional alcohols in excess such as ethylene glycol, diethylene glycol, and neopentyl glycol. Polyesters are commonly used in food packaging materials, such as polyethylene terephthalate (PET), and many monomeric raw materials have previously been fully evaluated for toxicity and are compliant with food packaging regulations [2]. However, there is not European, harmonized legislation specified to date for varnishes and coatings for food contact. There is only one specific regulation in order to control bisphenol A diglycidyl ether (BADGE), as well as its hydrolysis and hydrochlorination products, in epoxy resins, among others [3].

Additionally, additives can be incorporated into the polyester formulation to provide the functionality that is necessary such as cross-linking agents, catalysts, lubricants, pigments, solvents, etc. [4,5]. During the manufacturing, some substances, such as monomers, additives, or oligomers, may remain unreacted in the polymeric network and migrate into the food [6]. The many possible combinations of the different monomers in polyester-based coatings provide a large number of possible oligomers that have the potential to migrate from can coatings into food [5]. These low-molecular-weight compounds, such as small linear or cyclic polyester oligomers, polyester hydrolysis products, and manufacturing byproducts of polyester coatings, are considered as non-intentionally added substances (NIAS) [2].

Several analytical methods have been proposed in the literature for the analysis of polyester composition, mostly applying liquid chromatography coupled to different detection techniques for acid compounds analysis and gas chromatography for alcohols after a hydrolysis, transesterification, or aminolysis step [5,6,7,8,9,10].

Migration tests are required to identify and quantify potential migrants in order to assess the risks and safety of these coatings. These migration tests are challenging because the formulation used in the manufacture of can coatings is typically unknown for most analytical laboratories, and NIAS, such as polyester oligomers, can migrate into the food simulant, increasing the complexity of the analyses since reference standards are not commercially available. Therefore, it is necessary to perform a previous, non-targeted analysis in order to collect as much information as possible about the coating formulation [2]. Some studies have been published reporting the migration of polyester monomers and oligomers from polyester can coatings in the literature [6,8,9,10], but there is very limited information on non-targeted analysis of this type of resin.

In this work, several analytical techniques were used in order to characterize the type of coating on the food-contact surface of the analyzed can samples. Firstly, infrared spectra were acquired using an ATR-FTIR spectrometer, and the identification of the polymer type was achieved by comparison with polymer spectrum libraries. Confocal Raman microscopy was also applied to provide a complete characterization of the coating layers of the samples. A non-targeted screening, including gas chromatography coupled to mass spectrometry (GC-MS) with a purge and trap (P&T) technique, headspace solid-phase microextraction (HS-SPME), and liquid injection analysis, were applied to detect volatile and semi-volatile compounds. Then, the samples were extracted with an organic solvent and analyzed by matrix-assisted laser desorption coupled to time-of-flight mass spectrometry (MALDI-TOF MS) in positive mode to investigate the potential non-volatile migrants. Data published in the scientific literature and the main monomers used in the manufacture of polyester coatings were used to create a homemade database of possible monomer combinations. This database was then used to tentatively identify some of the compounds used in the manufacturing. Finally, migration solutions were obtained and analyzed by liquid chromatography coupled to ion trap mass spectrometry (LC-MS^n^) in order to investigate the presence of monomers in the polyesters by a targeted analysis in MS^n^ mode and to tentatively identify possible oligomers below 1000 Da in a full-scan analysis.

## 2. Materials and Methods

### 2.1. Reagents and Standards

Acetonitrile (ACN) HPLC grade, methanol (MeOH) GC-MS grade, absolute ethanol (EtOH) for analysis, formic acid 98–100% for analysis, butanol for analysis, and toluene for analysis were provided by Merck (Darmstadt, Germany). Acetonitrile LC-MS grade was purchased from Scharlau (Barcelona, Spain). Ultrapure water was prepared using a Direct-Q system (Millipore Ibérica, S.A., Madrid, Spain).

Analytical standards of polyvalent carboxylic acids and polyols were used for confirmation purposes: phthalic acid ≥ 99.5% (PA, CAS 88-99-3), isophthalic acid ≥ 97% (IPA, CAS 121-91-5), terephthalic acid ≥ 98% (TPA, CAS 100-21-0), adipic acid ≥ 99.5% (AA, CAS 124-04-9), trimellitic acid ≥ 99% (TMA, CAS 528-44-9), 1,4-cyclohexanedimethanol mixture of cis and trans 99% (CAS 105-08-8), neopentyl glycol 99% (CAS 126-30-7), ethylene glycol anhydrous 99.8% (CAS 107-21-1), 1,6-hexanediol ≥ 96.5% (CAS 629-11-8), diethylene glycol ≥ 99.5% (CAS 111-46-6), and 2-methyl-1,3-propanediol 99% (CAS 2163-42-0) were purchased from Sigma-Aldrich (Schnelldorf, Germany). 1,3-propanediol ≥ 98% (CAS 504-63-2) was obtained from Merck (Darmstadt, Germany).

Other analytical standards used for identification, 2-butoxyethanol ≥ 99%, hexanal, diisobutyl phthalate 99%, dibutyl phthalate 99%, methyl palmitate 97%, 2,4-di-tert-butylphenol 99%, 1-(benzyloxy)naphthalene, caprolactam ≥ 99%, octocrylene 97%, squalene ≥ 98%, and hexamethylenetetramine 99%, were purchased from Sigma-Aldrich (Schnelldorf, Germany). Nonanal 98.7% was obtained from Supelco (Bellefonte, PA, USA). Phenol ≥ 99.5% and dimethyl isophthalate 99% were provided by Merck (Darmstadt, Germany). Bisphenol A ≥ 99% was obtained from Aldrich-Chemie. Bis(2-ethylhexyl) adipate ≥ 99%, bis(2-ethylhexyl)phthalate 99%, and acetyltributyl citrate 99% were purchased from Fluka (Steinheim, Germany).

Standard solutions of each carboxylic acid (50 mg/L) were individually prepared in acetonitrile, except for terephthalic acid which was prepared in methanol. Regarding polyols, a stock solution at a concentration of 1000 mg/L was prepared in ethanol for each one. Working solutions of all compounds were prepared by subsequent dilutions and stored at 4 °C.

### 2.2. Samples and Extraction Procedures

The three can samples (CM1, CM2, and CM3) used in this study were provided by industrial partners. All samples were provided as coated tinplate sheets intended for use as food-contact materials. However, no detailed information was available from the suppliers on the type of coating and on the chemical composition of the lacquers used for the coating production. One of the samples (CM2) presented a white coating on the inner side and ink printing on the outer side, while the other two had a gray coating and a bright yellow coating on the inner and outer sides, respectively (CM1 and CM3).

#### 2.2.1. Volatile and Semi-Volatile Compounds Extraction Methods

For the analysis of potential volatiles, 5 g of each sample, previously cut into small pieces, was extracted using a Teledyne Tekmar Stratum Purge and Trap (P&T) system (Teledyne Tekmar, Mason, OH, USA) controlled with the VOC TekLink 3.2 software. The experimental conditions of the P&T were as follows: VocarbTM 3000 trap, sample temperature of 90 °C, purge flow of 40 mL/min, purge time of 30 min, desorb time of 2 min, desorb temperature of 250 °C, and desorb flow of 400 mL/min.

For volatile and semi-volatile analysis, different extraction solvents were tested, including ACN, EtOH, and MeOH. A known surface of each coated tinplate sheet (1 dm^2^) was extracted by immersion with 10 mL of solvent at 70 °C in an oven for 24 h. Then, the whole extract was concentrated under a nitrogen stream (RapidVap Vertex Evaporator, Labconco) to 0.5 mL, vortexed, and filtered through a polytetrafluoroethylene (PTFE) 0.22 µm filter (Advantec, Tokyo, Japan) for GC analysis.

For the HS-SPME technique, 1 g of each sample, previously cut into small pieces, was weighted into headspace vials with PTFE septa (20 mL; Agilent Technologies) and duplicated. HS-SPME injection was carried out with a SPME fiber assembly divinylbenzene/carboxen/polydimethylsiloxane (DVB/CAR/PDMS) from Supelco (Bellefonte, PA, USA). Prior to use, the fiber was conditioned for 1 h at 270 °C following the supplier’s instructions. The volatile compounds were exposed to the fiber at 80 °C for 30 min. Then, the fiber was inserted into the injector port for thermal desorption for 10 min at 200 °C, and the compounds were separated by GC and were identified using the MS detector operating in the conditions described below. Then, the fiber was baked at 270 °C for 5 min.

#### 2.2.2. Non-Volatile Compounds Extraction Method

For non-volatile analysis, migration cells from Triskelion (Zeist, The Netherlands) were used. A known surface of the food-contact side of each coated tinplate sheet (2.34 dm^2^) was extracted with 200 mL of ACN at 70 °C in an oven for 24 h. Then, an aliquot of the extract was submitted to MALDI analysis.

### 2.3. Migration Tests

Migration tests of the internal side were carried out in all samples (in duplicate). The test conditions applied were 4.5 h in an oven at 60 °C using ethanol 95% (v/v) as food simulant. The contact surface was 2.34 dm^2^, and the cells were filled with 200 mL of the simulant. After the incubation time, the migration cells were removed from the oven and left to cool down to room temperature. An aliquot was filtered through a PTFE 0.22 µm filter prior to the analysis by liquid chromatography.

### 2.4. Equipment Instrumental Analysis

#### 2.4.1. Fourier-Transform Infrared Spectroscopy (FTIR)

To identify the type of polymeric coating, infrared spectra were acquired using an ATR (attenuated total reflectance)-FTIR spectrometer (ATR-PRO-ONE, FTIR 4700, Jasco, Tokyo, Japan) equipped with a diamond optical crystal in the region from 4000 to 650 cm^−1^. The analysis was done on both surfaces, internal and external side, of each sample by covering the entire crystal surface. The ATR-FTIR spectrometer was controlled by the software Spectra Manager (version 2), and the identification was performed by using the KnowItAll 17.4.135.B software to compare the sample spectra obtained with several commercial databases related to polymers (IR Spectral Libraries of Polymers & Related Compounds from Bio-Rad Laboratories, Inc., Philadelphia, PA, USA).

#### 2.4.2. Confocal Raman Microscopy

Measurements were performed using a WITec confocal Raman microscopy alpha300 R (WITec GmbH, Ulm, Germany) coupled to an Ultra-High-Throughput-Spectrometer UHTS300 for visible equipped with a back-illuminated, charge-coupled device (CCD) camera with a quantum efficiency >90% (500–700 nm). The chip dimensions of the camera were 1024 × 127 pixels with pixel dimension of 26 × 26 µm^2^. The excitation source was a diode laser with an emitting wavelength of 532 nm. Laser power was adjusted to 8 mW. Raman spectra were recorded with a 50× objective (Epiplan Neofluar N.A. 0.8, Zeiss EC, Germany) in the range of 0 to 3600 cm^−1^. A grating that covers the full Raman spectrum was used (600 g/mm). The data were processed using WITec Project Five 5.1 software (WITec GmbH, Ulm, Germany). Each sample was investigated by performing an x-z scan with a scan range of 90 × 30 µm^2^, 140 × 60 pixels (8400 spectra), and 1 ms/spectrum acquisition time. The spectra identification was performed by using WITec True Match Database Management software to compare the sample spectra obtained with the commercial database of polymers and polymer additives from ST Japan.

#### 2.4.3. Gas Chromatography (GC)

The P&T GC-MS analysis was carried out using a Finnigan Trace Gas Chromatograph Ultra with a Finnigan Trace DSQ mass detector from Thermo Scientific (Fremont, California, USA). The volatile compounds were separated on a Rxi-624Sil MS (30 m × 0.25 mm internal diameter, 1.40 µm film thickness) column from Restek^®^ (Bellefonte, PA, USA). The chromatographic conditions were as follows: helium was used as carrier gas at a constant flow rate of 1 mL/min; the oven program was initially set at 45 °C for 4 min, then increased at a rate of 8 °C/min until 250 °C and held for 5 min; the transfer line and source temperature were set at 250 °C and 200 °C, respectively. The mass spectra were obtained with a mass-selective detector operated under electron impact ionization mode at a voltage of 70 eV, and data acquisition was performed in full-scan mode over an *m*/*z* range of 20–500. For data acquisition and processing, Xcalibur 2.0.7 software was used. Compounds were identified using the commercial mass spectral libraries NIST/EPA/NIH 11 (version 2.0) and Wiley Registry^TM^ 8th edition.

For the HS-SPME technique, an Agilent 6890 gas chromatograph equipped with an Agilent 5975B mass spectrometer (Agilent Technologies, Santa Clara, CA, USA) and a Combi-Pal autosampler (CTC Analytics, Zwingen, Switzerland) was used. The chromatographic capillary column was an HP-5MS 5% phenyl methyl from Agilent (25 m × 0.25 mm × 0.25 µm). The injection was performed in splitless mode, and helium was used as carrier gas at a constant flow rate of 1 mL/min. The oven program was initially set at 40 °C for 2 min, then increased at a rate of 9 °C/min until 300 °C and held for 10 min. The transfer line and source temperature were set at 320 °C and 230 °C, respectively. The chromatograms were acquired in full-scan mode over an *m/z* range of 20–500. Compounds were identify using the commercial mass spectral libraries NIST2014 and Wiley Registry^TM^ 8th edition.

For the analysis of semi-volatile compounds, a Thermo Scientific Trace 1300 Series gas chromatograph with a Trace ISQ LT mass spectrometer detector and an AI 1310 autosampler injector was used to carry out the GC analysis (Thermo Fischer Scientific, San José, CA, USA). The chromatographic conditions were as follows: an Rxi-5Sil MS 5% diphenyl, 95% dimethyl polysiloxane (30 m × 0.25 mm × 0.25 µm) column from Restek^®^ (Bellefonte, PA, USA) was used; the injection port temperature was set at 300 °C. The injection mode was splitless, and the injection volume was 1.0 µL. Helium was used as carrier gas at a constant flow rate of 1 mL/min. The transfer line and source temperature were set at 300 °C. The oven temperature program was the same as the one used for the analysis by HS-SPME. The chromatograms were acquired in full-scan mode over an *m/z* range of 35–500. The mass spectra were obtained with a mass-selective detector under electron impact ionization mode at a voltage of 70 eV. For data acquisition and processing, Xcalibur 3.0.63.3 software was used. Mass spectra libraries NIST/EPA/NIH 11 (version 2.0) and Wiley Registry^TM^ 8th edition were used for identification purposes.

#### 2.4.4. Matrix-Assisted Laser Desorption coupled to Time-of-Flight Mass Spectrometry (MALDI-TOF MS)

The MALDI-TOF MS analyses were performed on an Autoflex III MALDI TOF-TOF from Bruker Daltonics (Bremer, Germany) with a smartbeam-I laser. All spectra were acquired in the positive-ion reflector mode at an accelerating voltage of 25 kV. Trans-2-[3-(4-tertbutylphenyl)-2-methyl-2-propenylidene]malononitrile (DCTB), 20 mg/mL, was used as MALDI matrix, sodium trifluoroacetate (NaTFA), 10 mg/mL, as the cationization agent, and tetrahydrofuran (THF) as dissolvent. For the analysis, the preparation made was matrix/sample/cationization agent (10:2:1). Bruker FlexControl software was used for data acquisition and Bruker FlexAnalysis software for data processing. Ions in the mass range (*m/z*) of 350−4000 were detected. Peptide calibration standard II from Bruker was used as external calibrant, enabling a mass accuracy of about 10 ppm.

#### 2.4.5. Liquid Chromatography Coupled to Ion Trap Mass Spectrometry (LC-MS^n^)

The migration extracts were analyzed by an LC-MS^n^ system that comprised an Agilent Technologies 1200 Series HPLC system (Waldbronn, Germany) equipped with a quaternary pump, a degassing device, an autosampler, a column thermostat system, and a diode array detector (DAD), all of them controlled by the ChemStation for LC 3D systems software, along with the Agilent 6330 Ion Trap mass spectrometry detector controlled by the 6300 Series TrapControl version 6.1 software.

The chromatographic separation was achieved on a Gemini C18 110Å (150 mm × 3 mm internal diameter, 5 µm particle size) column with a pre-column from Phenomenex^®^ (Torrance, CA, USA). Mobile phases were 0.1% formic acid in ACN and 0.1% formic acid in water. A gradient elution method was applied, starting at 20% can, and this percentage gradually increased, reaching 50% at minute 25; this composition was held constant until minute 45, then the can concentration was linearly increased, reaching 100% at 60 min, and was held constant until minute 70. The column was kept at 35 °C and the autosampler was maintained at ambient temperature. The flow rate remained constant at 0.4 mL/min, and the injection volume was 10 µL.

The conditions of the ion trap mass spectrometer for the identification of unknown polyester oligomers were the following: positive electrospray ionization (ESI) mode, dry temperature was set at 350 °C, nitrogen was used as the nebulizer gas at a pressure of 15 psi, dry gas at 10 L/min, HV capillary at 3000 V, MS data were acquired in full-scan mode using two ranges (100 to 500 *m*/*z* and 500 to 1000 *m*/*z*), averages of 5 spectra, maximum accumulation time of 200 ms. The fragmentation was carried out in SmartFrag mode with a ramped collision energy applied over a range of 0.3 to 2.0 V. Helium was used as collision gas. The fragmentation spectra were obtained, and the proposed structures were checked through ACD/MS Fragmenter (v. 11.03) software.

In addition, a targeted analysis was carried out in MS(n) in negative ESI mode for the simultaneous determination of five polyvalent carboxylic acid monomers, including phthalic acid (PA), isophthalic acid (IPA), terephthalic acid (TPA), adipic acid (AA), and trimellitic acid (TMA). The *m*/*z* values selected were *m*/*z* 164.8 for PA, IPA and TPA, *m*/*z* 145.0 for AA, and *m*/*z* 208.8 for TMA.

## 3. Results and Discussion

### 3.1. FTIR-ATR and Confocal Raman Microscopy Analysis

The FTIR results allowed the identification of the type of coating present in the can samples of this study, on both the internal and external side. Only the best matches with the libraries were selected with a high quality index (HQI) higher than 90.

**Figure 1:**
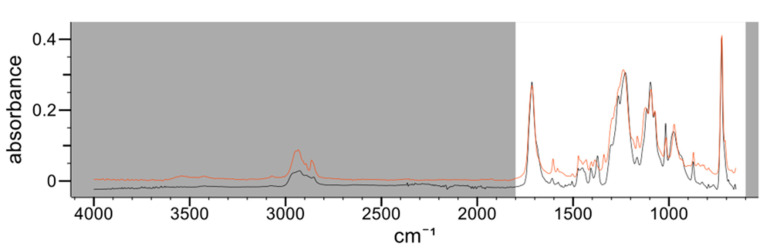
IR spectrum corresponding to the internal side of the sample CM2 (black line) compared to the first entry of the spectral libraries corresponding to the composite polyester spectrum (red line). Absorbance vs. wavenumber (cm^−1^).

**Figure 2:**
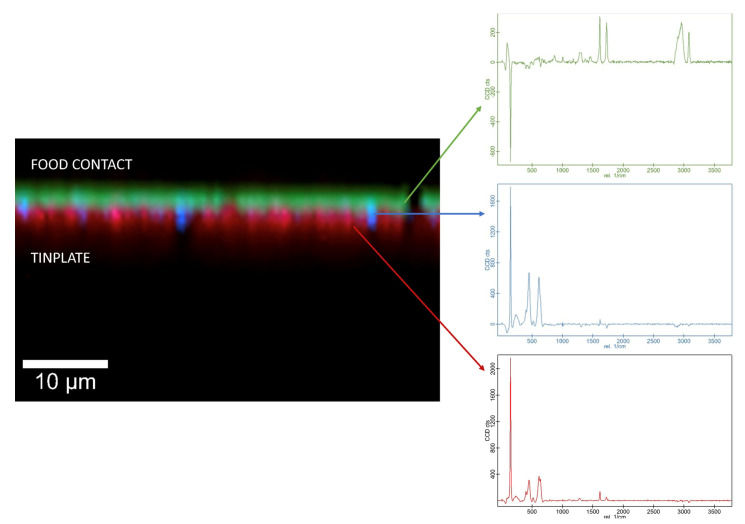
Raman spectra and corresponding, color-coded Raman image of the internal side of the sample CM2. Green: PET; blue: titanium oxide; red: polyester.

In the three samples, an internal, polyester-type coating was identified. In sample CM2, a composite spectrum with two types of polyester coating could be differentiated with an HQI of 96.17; one of them being identified as polyethylene terephthalate (PET). This identification coincides with the bibliography since the photoactive layer of TiO_2_, responsible for the white color, could be applied to the polymer to prevent oxidation of the PET [11]. Figure 1 shows the IR spectrum on the internal side of the sample CM2 (black line) overlaid with the first entry of the IR spectral libraries corresponding to the composite spectrum (red line). In addition, this identification could be confirmed by confocal Raman microscopy. This technique provided a complete characterization of the coating layers by imaging the different layers, and it was confirmed that the layer in contact with the food was PET (Figure 2).

Polyester resins are produced by condensing an acid with one or more alcohols, followed by copolymerization with one or more cross-linking agents [12]. For example, PET is produced in an esterification reaction where ethylene glycol reacts with terephthalic acid. PET is a linear, transparent thermoplastic polymer which has the capacity to crystallize under certain, controlled conditions [13].

Regarding the external side, the bright yellow coating of samples CM1 and CM3 was identified as epoxy resin, while, in sample CM2, with an ink printing, styrene/acrylic copolymer was identified. Due to recent calls to try to eliminate commonly used epoxy resins, they are gradually being phased out as internal coatings for food-contact materials, and alternatives are being sought, such as polyester resins. Acrylic resins are used principally for external can coatings [12], providing good color retention and heat stability [14].

### 3.2. GC Analysis

#### 3.2.1. P&T GC-MS

Regarding the analysis of potential volatiles by dynamic headspace (P&T) coupled to GC-MS, several aldehydes (butanal, hexanal, octanal, nonanal, decanal), alcohols (isobutanol, butanol), and aromatic compounds (composed of at least one benzene ring in their structures) were detected, as shown in Table 1. Six of the substances were confirmed by injection of the respective standard, while the others were tentatively identified using the mass spectra libraries. Only the best matches with the libraries were selected (direct matching factors and reverse search matching higher than 800). It is important to consider that, in the GC tests, the analysis of the material included both sides, internal and external.

Some of the solvents used in coating formulations were identified in the samples such as toluene, 2-butanone, 2-butoxyethanol, and 2-butoxyethyl acetate.

Several compounds with an alkylbenzene and benzene (*m*/*z* 119, 134) structure were detected, which is in agreement with the type of compounds detected in thermoset polyester samples analyzed by Gramshaw et al. [15]. Xylenes and ethylbenzene are aromatic isomers with the chemical formula of C_8_H_10_ (*m*/*z* 91, 106) used to synthesize plasticizers and polyesters. The xylenes consist of three isomers: o-xylene (OX; CAS 95-47-6), m-xylene (MX; CAS 108-38-3), and p-xylene (PX; CAS 106-42-3), which differ in the positions of the two methyl groups on the benzene ring. PX is first oxidized to terephthalic acid or dimethyl terephthalate, OX is oxidized to phthalic anhydride, and MX is oxidized to isophthalic acid, which are the base of polyester resins [16]. Diethylbenzenes, such as 1,4-diethylbenzene found in sample CM2, are used as solvent and precursors for cross-linking agents in producing resins such as polyesters [17]. Compounds with trimethylbenzene structures (*m*/*z* 105, 120) were also detected in all samples. 1,2,4-trimethylbenzene (CAS 95-63-6), 1,3,5-trimethylbenzene (CAS 108-67-8), and 1,2,3-trimethylbenzene (CAS 526-73-8) are used to produce 1,2,4-benzenetricarboxylic acid (trimellitic acid, TMA), 1,3,5-benzenetricarboxylic acid (trimesic acid), and 1,2,3-benzenetricarboxylic acid (hemimellitic acid), respectively, by aerobic oxidation. These acids are used to make plasticizers and as monomers for polyester and polyamide resins as they have high thermal resistance [18].

Cyclohexanone, detected in sample CM1, together with cyclohexanol, is oxidized with nitric acid to produce adipic acid (AA, also called 1,6-hexanedioic acid), an intermediate in the production of polyester resins [19]. This information was very useful to try to find out which monomers were used in the manufacture of the polyester resins in the different samples analyzed.

Dimethyl glutarate, identified in sample CM1 and CM2, is a dicarboxylic acid ester used in paints, enamel, varnish, lacquer, thinner, paint stripper, remover, polyamide, polyester, resins, and plasticizers [20].

Methyl methacrylate, found in CM2, is a monomer used to produce acrylic resins and is listed in the positive list for monomers used for plastics coming into contact with foodstuffs with a group specific migration limit (SML) of 6 mg/kg [21]. This makes sense since a styrene/acrylic copolymer was identified in the outer side by FTIR-ATR. As regards the monomers used in the manufacturing process, the former Scientific Committee on Food evaluated methyl methacrylate and butyl methacrylate, allocating a group TDI (tolerable daily intake) of 0.1 mg/kg body weight per day (expressed as methacrylic acid) [22]. Methyl methacrylate is also used as an alternative cross-linking agent in styrene [23]. The NIAS 2-ethyl-1-hexanol, found also in this sample, could be an impurity from the monomer 2-ethylhexylacrylate which is used in the production of acrylic adhesives [24] and was also described as a product formed by thermal decomposition or hydrolysis of plasticizers such as bis(2-ethylhexyl) phthalate (DEHP) or bis(2-ethylhexyl) adipate (DEHA) [25].

**Figure 3:**
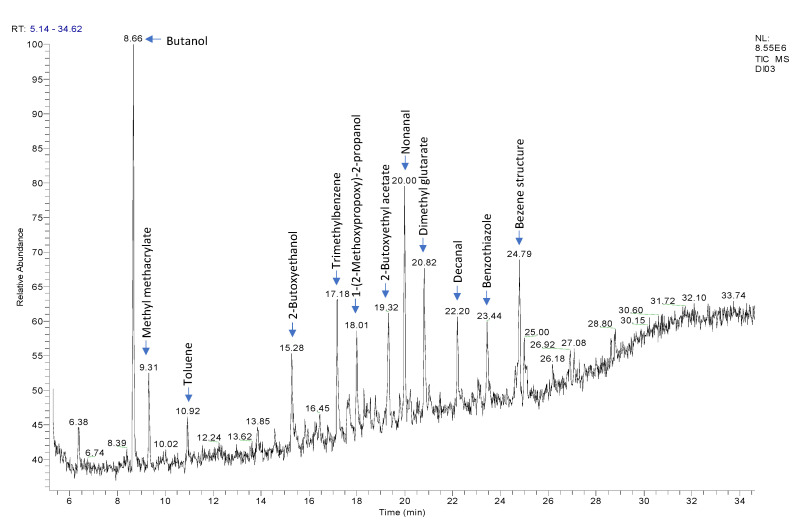
P&T GC-MS chromatogram of the coated tinplate sheet CM2 with the identification of some peaks.

In sample CM2, more compounds were detected, such as 1-(2-methoxypropoxy)-2-propanol, which are used as a solvent in the manufacture of water-based coatings and as a coalescing agent for water-based paints and inks [26]: phenol, which suggests that the main coating could undergo cross-linking reaction with a phenolic resin [27]; acetophenone, which could be produced during the heat degradation of PET [28], identified by FTIR-ATR for this sample; and benzothiazole, a photolytic decomposition product of an ultraviolet (UV) photoinitiator, which can be considered as an NIAS [29]. These photoinitiators are used to initiate polymerization during ink curing. For illustration, Figure 3 shows a GC chromatogram of the coated tinplate sheet CM2 with the identification of some peaks.

#### 3.2.2. Semi-Volatile Compounds by GC-MS

Regarding the analysis of potential semi-volatiles by GC-MS, several compounds were identified, including citrates, phthalates, adipates, alkanes, aldehydes, carboxylic acids, alcohols, diisocyanates, and fatty acids, among others. Different extraction solvents were tested, including ACN, EtOH, and MeOH. Since more peaks were observed when methanol was used as extraction solvent, only results obtained with this solvent are reported. Fourteen of them could be confirmed by injection of the respective standards, while the others were tentatively identified using the mass spectra libraries. Only compounds with appropriate direct matching factors (SI) and reverse search matching (RSI) were included in Table 2. The standards corresponding to the polyols mentioned in Section 2.1 were injected under the same conditions by GC-MS, but none of them was detected in the samples.

Polyester-type polymers result from a condensation reaction between polycarboxylic acids and polyols. The first benzene polycarboxylic acid to become a commercial product was phthalic acid. Phthalic anhydride, the commercial form of phthalic acid, was used in the manufacture of plasticizers, unsaturated polyesters, and alkyd resins and is obtained by the catalytic vapor-phase air oxidation of o-xylene or naphthalene. Terephthalic acid is used almost exclusively for the manufacture of PET. Another isomeric form, the dimethyl isophthalate, can be also used as monomer in the manufacture of polyesters, as well as the isophthalic acid [30]. Another common starting substance in polyesters manufacturing is adipic acid, which was identified in sample CM2. Several esters of the adipic acid were detected in this sample, but their identification with the libraries was not possible.

Related to the PET layer identified in sample CM2, the analytes diphenyl glycol and methyl-(2-hydroxyethyl) terephthalate were detected. Diphenyl glycol was also identified in extracts of multilayer films which contain PET in their composition [31], while methyl-(2-hydroxyethyl) terephthalate is a product formed in the depolymerization of PET into dimethyl terephthalate and ethylene glycol [32]. The compound terephthalic acid ester of neopentyl glycol cyclic dimer was also identified in all the samples. These short chain cyclic polyesters can be considered as NIAS and, therefore, a chemical standard is not commercially available to carry out safety evaluation studies [2].

Dimethyl adipate and dimethyl maleate, identified in sample CM2, can be used as monomers in the synthesis of polyesters via polycondensation [33], while methyl palmitate, detected in all the samples, is used as intermediate for detergents, emulsifiers, stabilizers, resins, lubricant, plasticizers, and defoamer in food-contact coatings [34]. 2,6-diisopropylnaphthalene, present in samples CM1 and CM2, is used for making monomers of high-performance polyester fibers, molded plastics, thermotropic liquid crystalline polymers, and other advanced polymer materials [35].

Several benzaldehyde compounds were identified in sample CM1, such as 2,5-dimethyl-4-methoxybenzaldehyde, 2-hydroxy-5-methylisophthalaldehyde, and 4-hydroxy-3,5-dimethylbenzaldehyde, which were also identified in the polyester coating analyzed by Putzu [36].

In addition to monomers, there are more chemical compounds involved in the manufacturing of polyester resins such as cross-linking agents, blocking agents, catalysts, lubricants, wetting agents, solvents, etc. Isophorone diisocyanate and caprolactam were present in the extracts; these substances are commonly used as a cross-linking and blocking agent, respectively, in polyester lacquers [37,38]. Both are included in the regulation (EU) 10/2011 [21]. Trimethylolpropane, which was detected in sample CM2, can be used as a cross-linking agent [39] or as a polyol in the alkyd synthesis for coating formulations [40]. It is included in the regulation (EU) 10/2011 with a specific migration limit (SML) of 6 mg/kg [21]. Benzoguanamine, identified in CM1 and CM2, is another, often cross-linking, agent used in saturated polyester resin for can coatings [41].

Different plasticizers, such as acetyl tributyl citrate, bis(2-ethylhexyl) adipate (DEHA), triphenyl phosphate, di(ethylene glycol) dibenzoate, and phthalates, were identified in the extracts. Diisobutyl phthalate is another plasticizer commonly associated with printing inks or adhesives [42]. Dibutyl phthalate and bis(2-ethylhexyl) phthalate (DEHP) were also detected. These compounds, as well as plasticizers, can be found in printing ink formulations and also have been employed as solvents to hold color [43]. Triphenyl phosphate is used as a plasticizer for coatings and lacquers, as well as a flame retardant and solvent [44,45], while di(ethylene glycol) dibenzoate is a plasticizer for polyvinyl chloride acetate and a component of adhesives [46].

Some degradation products from antioxidants used as additives were identified in the extracts, such as 1,3-di-tert-butylbenzene and 2,4-di-tert-butylphenol, which were described as degradation from Irgafos 168 and Irganox 1076 [47] or 7,9-di-tert-butyl-1-oxaspiro(4,5)deca-6,9-diene-2,8-dione, which is a degradation product of Irganox 1010 [44].

In sample CM2, compounds related with the acrylic resins were detected, such as isobornyl methacrylate and methyl methacrylate. Poly(methyl methacrylate) is a kind of acrylic resin, which is essentially produced by polymerizing a methyl methacrylate monomer [48]. However, in samples CM1 and CM3, where the outer coatings were identified as epoxy resins by FTIR-ATR, some compounds related with epoxy resins were identified. For example, the epoxy hardener hexamethylenetetramine [49] or the common monomer bisphenol A (BPA).

1-(benzyloxy)naphthalene and methyl dehydroabietate were found in sample CM1. The first is an UV-active sensitizer, a substance that acts as a kind of solvent for the reactants of the coloring process [50], while methyl dehydroabietate is a component of varnishes and printing inks used as a tackifier for the enhancement of adhesive performance [44,46]. Octocrylene, detected in sample CM3, is used as UV light absorber to prevent degradation of polymers. Squalene, identified in all the samples, has oxygen-scavenging capacity to extent the shelf life of oxygen-sensitive products [45].

Methyl benzoate and benzoic acid, identified in sample CM2, were described as photolytic decomposition products of the UV photoinitiator 2-hydroxy-2-methylpropiophenone, so they can be considered as NIAS. 2,4,6-trimethylbenzaldehyde, methyl 2,4,6-trimethylbenzoate, and 2,4,6-trimethylbenzoic acid were also degradation products of the photointiatior diphenyl(2,4,6-trimethylbenzoyl)phosphine oxide [29]. 1-dodecene is an olefin monomer [51], detected in sample CM2. 1-dodecene is included in the regulation (EU) 10/2011 with a specific migration limit (SML) of 0.05 mg/kg [21].

Some fatty acids were detected, such as: tetradecanoic acid, a lubricant and processing aid, included in the regulation (EU) 10/2011 [21]; pentadecanoic acid, an adhesive and potential resulting compound from the thermal oxidation of polyethylene [52]; and nonanoic acid used in lacquers, plastics, and as plasticizer [53]. Fatty acids are also used in the preparation of alkyds, which are oil-modified polyesters [54,55].

#### 3.2.3. HS-SPME-GC-MS

Some of the compounds identified by the HS-SPME technique (Table 3) were described previously, since they were detected in the analysis carried out with the other techniques by GC-MS. Several aldehydes (heptanal, octanal, nonanal, decanal, 2-decenal, 2-undecenal, tetradecanal, and 14-octadecenal), alkanes (undecane, dodecane, tridecane, tetradecane, and heptadecane), and acids (octanoic acid, nonanoic acid, and decanoic acid) were identified in the samples.

This technique made it possible to identify the neopentyl glycol monomer and glycerol in all samples analyzed, which are frequently used in the synthesis of polyester resins.

Pentaethylene glycol and hexaethylene glycol, present in sample CM3, are members of the homologous series of polyethylene glycols commonly used as raw materials in a large number of industrial applications such as the condensation with dimethyl terephthalate or terephthalic acid resulting in a polyester resin [56]. Dimethyl succinate, identified in the same sample, is a starting reactant used in the synthesis of some polyesters [57].

Ethyl acrylate was present in sample CM2, which presented a coating of acrylic resin according to the IR results. 2-ethylhexylacetate, also identified in this sample, could be an impurity from the commercial 2-ethylhexylacrylate, a monomer used in the production of acrylic adhesives [24].

1-butoxy-2-propanol and trans-2-nonenal, detected in samples CM1 and CM2, are compounds used in inks [58,59]. Other compounds related to inks, detected in all tested samples, were diethylene glycol monobutyl ether and 2-phenoxyethanol [60].

Some plasticizers were detected, such as benzyl alcohol [61], identified in sample CM3, and 2,2,4-trimethyl-1,3-pentanediol diisobutyrate [62], detected in all samples. Diethyl phthalate is another plasticizer widely used in resins, polymers, adhesives, paints, and lacquers and is also used as a solvent to hold color. Isopropyl myristate is used as plasticizer for cellulosic, as well as pigment dispersant and binder [45].

2-hydroxy-2-methylpropiophenone is a UV photoinitiator detected in sample CM2 [29]. 4-tert-butylphenyl glycidyl ether, which was present in sample CM3, is a reactive diluent used to reduce the viscosity of epoxy resins in order to improve polymerization [63].

### 3.3. MALDI-TOF MS

The samples were extracted and analyzed by MALDI-TOF MS technique. In order to tentatively identify the possible oligomers, a homemade database with the common starting monomers used in the formulations of polyester coatings was developed. The homemade database was created considering nineteen polyols (one monool, seventeen diols, and one triol) and six polyacids (five diacids and one triacid) known to be potentially used as monomers in polyester formulations intended for food-contact coatings, according to the literature. For each combination, the linear and fully cyclized forms (depending on the number of H_2_O losses) were considered, resulting in a high number of oligomer structures.

Observing the mass spectra obtained with MALDI-TOF MS, it was possible to notice that samples CM2 and CM3 presented a similar pattern, different from sample CM1. All of them presented series of signals appearing at intervals of 234.1 Da, which corresponds to a phthalic/isophthalic/terephthalic acid (PA) with neopentyl glycol (NPG), as reported in the study of Arnould et al. [64].

**Figure 4:**
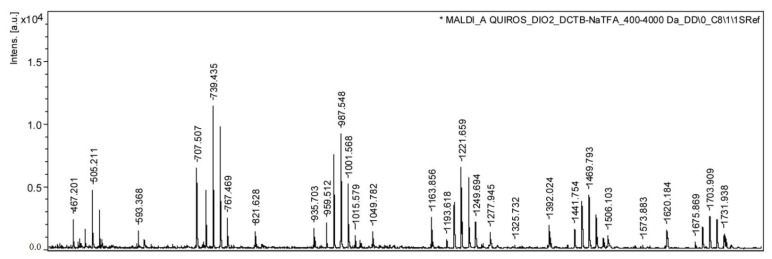
MALDI mass spectrum of the acetonitrile extract of sample CM1: intensity [a.u.] vs. *m/z*.

In sample CM1, adjacent mass groups were observed, separated by 14 Da intervals. Exchanging neopentyl glycol with 1,6-hexanediol (HD) increased the mass by 14 Da, while exchanging neopentyl glycol with 1,3-butanediol, 1,4-butanediol (BD), or 2-methyl-1,3-propanediol (MPO) reduced the mass by 14 Da. Taking into account the intensity of the masses, it can be concluded that the alcohol used in the highest proportion was NPG, followed by HD and BD/MPO. Within the first group of masses detected, it was possible to tentatively identify the adduct of the cyclic oligomer 2PA+2NPG with sodium (491.2), the adduct of the cyclic oligomer 2PA+NPG+HD with sodium (505.2), and the adduct of the cyclic oligomer 2PA+2HD with sodium (519.2). In addition, another series of signals appeared with intervals of 114.1 Da and were identified in this sample, which were assigned to the adduct of caprolactone cyclic oligomers with sodium. Caprolactone is used as an additive in polyester lacquers [38]. For illustration, as an example, the masses corresponding to 593.4, 707.5, and 821.6 were assigned to caprolactam cyclic pentamer, hexamer, and heptamer. Figure 4 shows the MALDI mass spectrum of the acetonitrile extract of this sample.

Regarding samples CM2 and CM3, adjacent mass groups were observed, separated by increments of 40 Da or reductions of 42 Da. Exchanging neopentyl glycol with 1,4-bis(hydroxymethyl)cyclohexane (CHDM) increased the mass by 40 Da, while exchanging neopentyl glycol with ethylene glycol (EG) reduced the mass by 42 Da. Taking into account the intensity of the masses, it can be concluded that the alcohol used in the highest proportion was NPG, followed by CHDM and EG in a lesser proportion. Within the first group of masses detected, the mass of greater intensity could be tentatively identified as the adduct of the cyclic oligomer 2PA+2NPG with a proton (469.2), as in the study of Omer et al. [65].

### 3.4. Analysis of Migration Tests by LC-MS^n^

As was seen in previous studies carried out by Paseiro-Cerrato et al. [6] and Pietropaolo et al. [38], the number of detected oligomers increased with the ethanol concentration. In our study, the most drastic conditions were simulated, and EtOH 95% was used to perform the migration tests.

In the present work, all combinations above 1000 Da were disregarded because it is generally recognized that compounds above this mass are not important from a toxicological point of view, based on the fact that they are not typically absorbed through the gastrointestinal tract. Moreover, when comparing the ionization mode, it was considered that the negative ionization mode did not bring any significant additional information since more intense, related signals were usually observed in the positive mode, in accordance with previous findings reported by Omer et al. and Paseiro-Cerrato and co-workers, who detected few linear oligomers in the negative mode [8,65]. Therefore, only the oligomers obtained in positive mode are displayed in Table 4.

The Cramer decision tree based on the molecular structure of the compound was used to estimate the toxicity of the identified compounds (Table 4). For this purpose, the software Toxtree v3.1.0 (Ideaconsult Ltd., Sofia, Bulgaria) was used. The Cramer scheme consists of 33 questions where each answer leads to another question or to a final classification into one of the three classes of toxicity: substances with simple chemical structures and for which efficient modes of metabolism exist are classified in class I (low toxicity), substances which possess structures that are less innocuous than class I but do not contain structural characteristics suggestive of toxicity like those substances in class III are classified in class II (intermediate toxicity), while class III (high toxicity) is assigned to substances with chemical structures that permit no strong initial impression of safety and may suggest a significant toxicity by having reactive functional groups [66].

Several homologous series (n = 3–8) of polyester oligomers were tentatively identified in the migration tests of the samples, more than those listed in Table 4, since the isomeric forms of the oligomers eluted at different retention times. These isomers can arise from using different isomers of starting substances (PA, IPA, TPA) or from oligomers having the same composition but different structures, taking into account that the position of acids and alcohols cannot be differentiated [5]. These results are in line with those reported by other authors. For example, two of them (2PA+CHDM and 2PA+2CHDM) were also detected in the extracts of the polyester can coatings analyzed by Paseiro-Cerrato et al. [8], while four of them (2TPA+2HD, 2PA+2NPG, 2PA+NPG+HD, and 3PA+3HD) could be quantified in one of the resins examined by Pietropaolo et al. [38], which had the same starting monomers as the one assumed for our samples. Omer et al. [65] analyzed two different polyester–polyurethane lacquers and predicted a total of twenty-eight oligomer combinations, of which eleven coincided with those identified in our samples. In agreement with the study carried out by Bradley et al. [5], most of the oligomers found were cyclic because they lacked free functional groups, hence, they could not be incorporated into the polymeric network of the coating and, therefore, could migrate as unreactive byproducts.

The exposure-based threshold of toxicological concern (TTC) is used to evaluate compounds with known chemical structure in terms of possible risks to human health. While linear polyester oligomers are mainly classified as Cramer I substances with a daily exposure threshold of 30 µg/kg body weight per day, it was observed that cyclic polyester oligomers formed from aromatic dicarboxylic acids are assessed as Cramer III substances, with a daily exposure threshold of 1.5 µg/kg body weight per day [10].

In addition, the fragmentation spectra were obtained when the adduct with hydrogen was the most intense in order to generate more information about the chemical structure of the proposed compounds (Table 4). When the sodium or ammonium adducts were formed and were the most intense, no fragmentation was observed, as also reported in the study of Úbeda et al. [67]. Some of the most repeated fragments were: *m/z* 149 corresponding to a phthalic acid (PA/IPA/TPA) with a loss of a water molecule and is protonated; *m/z* 235, which corresponds to a protonated molecule of phthalic/isophthalic/terephthalic acid (PA) with neopentyl glycol (NPG); *m*/*z* 383 corresponding to a molecule formed by two NPG with a PA, protonated [64]; *m*/*z* 193, which corresponds to PA with EG, protonated; *m/z* 359, which corresponds to two molecules of PA with one of EG, protonated; and *m/z* 415, which corresponds to two molecules of PA with one of HD, protonated.

There is not toxicological data available for these polyester oligomers and, therefore, no migration limits have been established in the legislation to date. Moreover, there is limited information available in the scientific literature on migration data regarding these migrants. Consequently, special attention should be paid to the safety of these compounds.

Regarding the targeted analysis for the simultaneous determination of five polyvalent carboxylic acid monomers, a chromatographic method was developed. Several columns, including C18 (100 mm × 2.1 mm, 2.6 µm), Eclipse XDB-C18 (100 mm × 4.6 mm, 1.8 µm), Narrow-Bore SB-C3 (150 mm × 2.1 mm, 5 µm), and Gemini C18 110Å (150 mm × 3 mm, 5 µm), were tested for the separation of the compounds, which is challenging in the case of isomers. Only Gemini C18 110Å (150 mm × 3 mm, 5 µm) allowed the separation of all the carboxylic acids.

None of them was detected in the migrations tests of the samples analyzed above the limits of detection (0.5 mg/L for PA, 0.1 mg/L for TPA, 0.25 mg/L for IPA and TMA, and 1.25 mg/L for AA), with the exception of TPA which was detected in sample CM1.

## 4. Conclusions

The analytical techniques presented in this study were very useful for the characterization and investigation of potential migrants from polyester-type coatings. Each technique provided complementary and valuable information. Firstly, coating materials were identified by ATR-FTIR spectrometer and confocal Raman microscopy. All samples presented an internal coating of polyester-type. To the best of our knowledge, this is the first time that confocal Raman microscopy has been used to characterize polymeric coatings, providing a characterization of the sample by imaging the different layers. Then, the use of GC-MS analytical methods made it possible, through a non-targeted screening, to identify potential volatile and semi-volatile migrant compounds present in polyester coatings. Several compounds were identified, including citrates, phthalates, adipates, alkanes, aldehydes, carboxylic acids, alcohols, diisocyanates, and fatty acids, among others. In addition, the signal series detected in the MALDI-TOF MS analysis, combining a homemade database with the common starting monomers used in the formulations of polyester coatings, allowed the elucidation of the starting substances used in the formulation of the samples analyzed, such as monomers and additives. Finally, the migration experiments carried out with ethanol 95% and subsequent analysis by LC-MS^n^ revealed the presence of various polyester oligomers with their corresponding fragmentation pattern. The results of this study confirm the complex task of trying to identify the potential migrants from coatings. For these chemical migrants there are no toxicological data available nor migration limits established to date. Consequently, a risk assessment is necessary for these compounds, which should not be underestimated.

## Figures and Tables

**Table 1:** Volatile compounds detected in the non-targeted analysis by P&T GC-MS.

TR (min)	CAS Nº	Compound	*m/z*	CM1	CM2	CM3
6.38	123-72-8	Butanal	44, 72	X	X	X
6.57	78-93-3	2-butanone	43, 72			X
7.69	78-83-1	Isobutanol	43, 74	X		
8.66	71-36-3	1-butanol *	41, 56	X	X	X
9.31	80-62-6	Methyl methacrylate	41, 69, 100		X	
10.92	108-88-3	Toluene *	65, 91	X	X	X
12.24	66-25-1	Hexanal *	43, 56			X
13.59	100-41-4	Ethyl benzene	91, 106			X
13.85		Xylene structure	91, 106	X	X	X
14.50		Xylene structure	91, 106	X	X	X
15.28	111-76-2	2-butoxyethanol *	57, 87	X	X	X
15.42	108-94-1	Cyclohexanone	55, 69, 98	X		
16.45		Trimethylbenzene	105, 120		X	
17.18		Trimethylbenzene	77, 105, 120	X	X	X
17.61	124-13-0	Octanal	41, 56, 69			X
17.99		Trimethylbenzene	59, 105, 120			X
18.01	13429-07-7	1-(2-methoxypropoxy)-2-propanol	59, 105, 120		X	
18.28	104-76-7	2-ethyl-1-hexanol	57, 70, 83		X	
18.57	105-05-5	1,4-diethylbenzene	105, 119, 134		X	
18.77	108-95-2	Phenol *	66, 94		X	
19.30		Unknown (benzene structure)	119, 134			X
19.32	112-07-2	2-butoxyethyl acetate	43, 57, 87	X	X	
19.79	98-86-2	Acetophenone	70, 105, 120		X	
20.00	124-19-6	Nonanal *	28, 32, 57, 70	X	X	X
20.26		Unknown (benzene structure)	119, 134		X	
20.82	1119-40-0	Dimethyl glutarate	59, 100, 129	X	X	
22.20	112-31-2	Decanal	28, 41, 57, 70	X	X	X
23.44	95-16-9	Benzothiazole	69, 108, 135		X	
24.79		Unknown (benzene structure)	119, 131, 147		X	
25.00		Unknown (naphthalene structure)	117, 131, 160		X	

* confirmed with standards.

**Table 2:** Semi-volatile compounds detected in the methanolic extracts analyzed by GC-MS.

TR (min)	CAS Nº	Compound	*m*/*z*	SI	RSI	CM1	CM2	CM3
9.34	104-76-7	2-ethyl-1-hexanol	43, 57, 79, 83	709	955		X	
10.56	93-58-3	Methyl benzoate	77, 105, 136	750	854		X	
11.03	1587-15-1	Dimethyl maleate	43, 71, 103	739	922		X	
11.77	65-85-0	Benzoic acid	51, 77, 105, 122	805	956		X	
12.17	112-41-4	1-dodecene	43, 55, 69, 83	934	935		X	
12.44	527-54-8	3,4,5-trimethylphenol	91, 121, 136	760	885		X	
12.95	627-93-0	Dimethyl adipate	101, 114, 143	700	818		X	
13.01	100-97-0	Hexamethylenetetramine *	42, 112, 140	617	905	X		
13.11	1014-60-4	1,3-di-tert-butylbenzene	41, 57, 91, 175	744	832			X
13.32	105-60-2	ε-caprolactam *	55, 85, 113	766	849	X		
13.38	112-05-0	Nonanoic acid	60, 73, 115	700	773		X	
13.50	629-62-9	Pentadecane	43, 57, 71, 85	765	867			X
13.55	77-99-6	Trimethylolpropane	41, 57, 71, 86	903	916		X	
13.75	627-91-8	Methyl adipate	55, 74, 100, 114, 129	713	882		X	
14.03	487-68-3	2,4,6-trimethylbenzaldehyde	91, 119, 147	859	946		X	
14.20	85-44-9 88-99-3	Phthalic acid pure or anhydride phthalic	50, 76, 104, 148	776	880		X	
14.56	2282-84-0	Methyl 2,4,6-trimethylbenzoate	91, 119, 147, 178	860	862		X	
14.88	124-04-9	Adipic acid	41, 55, 87, 100	725	823		X	
15.83	480-63-7	2,4,6-trimethylbenzoic acid	119, 146, 164	900	902		X	
15.95	6745-75-1	2,5-dimethyl-4-methoxybenzaldehyde	77, 163, 164	744	765	X		
16.09	7310-95-4	2-hydroxy-5-methylisophthalaldehyde	77, 90, 107, 136, 164	825	831	X		
16.44	7534-94-3	Isobornyl methacrylate	41, 69, 95, 136	927	928		X	
16.51	2233-18-3	4-hydroxy-3,5-dimethylbenzaldehyde	77, 91, 121, 149, 150	892	918	X		
16.63	487-69-4	2,4-dihydroxy-6-methylbenzaldehyde	106, 123, 151	714	745	X		
16.95	96-76-4	2,4-di-tert-butylphenol *	57, 191, 206	781	834	X	X	X
17.03	1459-93-4	Dimethyl isophthalate *	76, 135, 163, 194	860	924	X	X	
17.66	143-07-7	Dodecanoic acid	43, 60, 73	853	901		X	
18.09	4098-71-9	Isophorone diisocyanate	81, 110, 123	703	791	X	X	
18.81		Unknown (acrylate structure)	55, 68				X	
19.18		Unknown (alcohol structure)	69, 83, 97	726	842		X	X
19.35	100-21-0 121-91-5	Terephthalic acid or isophthalic acid	65, 120, 149, 166	716	753		X	
19.46		Unknown (alkane structure)	57, 71, 85			X		X
19.82	24157-81-1	2,6-diisopropylnaphthalene	155, 197, 212	793	834	X	X	
19.89		Unknown (ester of adipic acid)	55, 111, 143				X	
20.19	544-63-8	Tetradecanoic acid	43, 60, 73, 129	883	893	X	X	
20.67	26896-48-0	Tricyclodecanedimethanol	67, 79, 91, 119, 147	826	832		X	
20.82	104-66-5	Diphenyl glycol	77, 121, 214	815	824		X	
21.16	502-69-2	6,10,14-trimethylpentadecan-2-one	43, 58, 71	908	939			X
21.20	3645-00-9	Methyl-(2-hydroxyethyl) terephthalate	76, 163, 181	832	907		X	
21.35	1002-84-2	Pentadecanoic acid	60, 73, 129	700	767	X	X	
21.39	84-69-5	Diisobutyl phthalate *	41, 57, 149	858	932	X	X	X
21.62		Unknown (alcohol structure)	69, 83, 97	846	897		X	
21.92	82304-66-3	7,9-di-tert-butyl-1-oxaspiro [4.5]deca-6,9-diene-2,8-dione	41, 57, 175, 205	709	709			X
22.10	112-39-0	Methyl palmitate *	43, 74, 87	891	932	X	X	X
22.16		Unknown (eicosene structure)	43, 55, 70, 83					X
22.27		Unknown (acid structure)	55, 69, 81, 96			X	X	
22.45	84-74-2	Dibutyl phthalate *	149	892	926			X
22.51	57-10-3	Hexadecanoic acid	43, 60, 73, 129	922	962	X	X	
22.58		Unknown (alcohol structure)	82, 95, 109					X
23.27		Unknown (phthalate structure)	56, 76, 163, 181					X
23.43	91-76-9	Benzoguanamine	43, 76, 103, 187	921	937	X	X	
23.46		Unknown (phthalate structure)	56, 163, 181					X
23.72		Unknown (phthalate structure)	149	747	766		X	
23.83		Unknown (alcohol structure)	43, 57, 69, 83, 97	851	939		X	
23.98	112-62-9	Methyl oleate	55, 69, 74, 83, 97	888	907	X	X	
23.98	629-92-5	Nonadecane	43, 57, 71	754	855			X
24.24	112-61-8	Methyl stearate	74, 87, 143	771	898	X	X	X
24.39		Unknown compound (octadecenoic acid structure)	55, 69, 83, 97	888	888	X	X	
24.64	57-11-4	Stearic acid	43, 57, 73	876	931	X	X	
24.78	607-58-9	1-(benzyloxy)naphthalene *	91, 65, 115, 234	717	819	X		
24.88	80-05-7	Bisphenol A (BPA) *	91, 119, 213	855	860	X		X
25.00		Unknown (alkane structure)	43, 57, 71, 85					X
25.42	77-90-7	Acetyltributyl citrate (ATBC) *	129, 185, 259	771	901	X		
25.63		Unknown (acetophenone structure)	77, 91, 119, 147	861	906		X	
25.72		Unknown (benzoic acid structure)	77, 105	735	892		X	
25.97		Unknown (isophthalic acid structure)	82, 149, 167, 205				X	
26.15		Unknown compound (octadecenoic acid structure)	55, 69, 85, 97	779	790	X		
26.17		Unknown (phenol structure)	121, 227, 256					X
26.45	1235-74-1	Methyl dehydroabietate	239, 299	700	711	X		
26.78	103-23-1	Bis(2-ethylhexyl) adipate (DEHA) *	57, 70, 111, 129	750	943	X		
26.89		Unknown (alkane structure)	57, 71, 85					X
26.96	115-86-6	Triphenyl phosphate	77, 94, 326	752	908		X	
27.57	10546-70-0	n-propylbenzamide	77, 105, 163	809	955	X	X	X
27.64	120-55-8	Di(ethylene glycol) dibenzoate	77, 105, 149	888	967	X		
27.78		Unknown (alkane structure)	43, 57, 71					X
28.07	117-81-7	Bis(2-ethylhexyl) phthalate (DEHP) *	149, 167	841	916	X	X	X
28.25		Unknown (isophthalic acid structure)	82, 149, 231			X		
28.39		Unknown (phthalate or benzoic acid structure)	149, 167, 235, 253			X	X	X
28.87		Unknown (phthalate or benzoic acid structure)	149, 167, 235					X
29.11	6197-30-4	Octocrylene *	178, 204, 248	613	840			X
29.75		Unknown (phthalate structure)	82, 104, 149, 383			X		
29.47		Unknown (alkane structure)	43, 57, 71					X
30.20		Unknown (phthalate structure)	82, 104, 149, 383			X		
30.39	111-02-4	Squalene *	69, 81	677	787	X	X	X
35.76		Unknown (ester of adipic acid)	55, 83, 129				X	
36.70		Unknown (phthalate structure)	83, 149				X	
38.36		Terephthalic acid ester of neopentyl glycol cyclic dimer (C_26_H_28_O_8_)	76, 104, 132, 149, 338, 383, 468	773	961	X	X	X

* confirmed with standards; SI: direct matching factor; RSI: reverse search matching factor.

**Table 3:** Volatile compounds detected in the samples analyzed by HS-SPME-GC-MS.

TR (min)	CAS Nº	Compound	*m*/*z*	SI	RSI	CM1	CM2	CM3
5.72		Xylene structure	91, 106	814	930	X	X	X
5.94	140-88-5	Ethyl acrylate	55, 27, 99	700	818		X	
6.1	108-94-1	Cyclohexanone	55, 42, 98, 69	893	913	X		
6.3	111-71-7	Heptanal	43,70,55	811	876	X	X	X
6.4	111-76-2	2-butoxyethanol	57,45,87	856	878	X	X	X
6.9	126-30-7	Neophentyl glycol	56,73,31	839	881	X	X	X
7.1	5131-66-8	1-butoxy-2-propanol	45,57,87	823	878	X	X	
7.6		Trimethylbenzene	105,12				X	
8.1		Trimethylbenzene	105,12	730	924	X	X	X
8.2	124-13-0	Octanal	41,57,84	749	944	X	X	X
8.5	584-03-2	1,2-butanediol	59,43,73	785	810	X	X	X
8.6–9.6	56-81-5	Glycerol	61,43,31	735	804	X	X	X
8.63		Trimethylbenzene	105, 120	767	848			X
8.7	104-76-7	2-ethyl-1-hexanol	57,41,70	764	877	X	X	X
8.8	106-65-0	Dimethyl succinate	115, 55, 87	789	943			X
8.84	100-51-6	Benzyl alcohol	79, 108	785	867			X
9.4	98-86-2	Acetophenone	105, 77, 120	704	827			X
9.5	103-09-3	2-ethylhexyl acetate	43,56,70	759	775		X	
9.5	111-87-5	1-octanol	56, 70, 83	768	891	X		X
9.6	3101-60-8	4-tert-butylphenyl glycidyl ether	191, 135, 206	700	683			X
9.8		Tetramethylbenzene	119,91,134			X	X	
9.86	112-07-2	2-butoxyethyl acetate	43,57,87	794	859	X	X	X
10.05	1120-21-4	Undecane	43,57,71,85	896	945	X	X	
10.1	124-19-6	Nonanal	41,57,29,70	948	948	X	X	X
10.4	488-23-3/527-53-7/934-74-7	Tetramethylbenzene	119,134	715	798	X	X	X
10.6	1119-40-0	Dimethyl glutarate	59,100,129	862	900	X	X	X
11.05	18829-56-6	Trans-2-nonenal	41, 55, 70	761	865	X	X	
11.4	124-07-2	Octanoic acid	60, 73, 43	836	894	X	X	X
11.55	112-34-5	Diethylene glycol monobutyl ether	45, 57, 29	717	917	X	X	X
11.6	112-41-4	1-codecene	43, 55, 69, 83, 97	836	894		X	
11.7	112-40-3	Dodecane	43, 57, 71, 85	932	933	X	X	X
11.8	112-31-2	Decanal	41, 57, 82, 95	948	951	X	X	X
11.99	87-61-6/120-82-1	Trichlorobenzene	180, 145, 109, 74	878	901	X	X	
12.07	122-99-6	2-phenoxyethanol	94, 138, 77	911	912	X	X	X
12.2	95-16-9	Benzothiazole	135, 108, 69	855	937	X	X	X
12.4	627-93-0	Dimethyl adipate	59, 114, 143	811	895	X	X	X
12.7	3913-81-3	2-decenal	41, 55, 70, 83	778	903	X	X	X
12.88	112-05-0	Nonanoic acid	55,41,73	739	847	X	X	X
13.1	7473-98-5	2-hydroxy-2-methylpropiophenone	57,77,105	739	837		X	
13.3		Unknown (methyl-naphthalene structure)	142, 115			X		
13.3	629-50-5	Tridecane	43,57,71	901	935		X	
13.4		Unknown (aldehyde structure)	41, 57, 68, 82	738	901	X		X
13.44		Unknown (benzaldehyde structure)	147,119,91	898	913		X	
13.8-13.9		Unknown (naphthalene structure)	131,160,145			X	X	
14.2	6846-50-0	2,2,4-trimethyl-1,3-pentanediol diisobutyrate	71,43,56,83	814	840	X	X	X
14.3	2463-77-6	2-undecenal	70,57,41	760	806	X	X	X
14.4	334-48-5	Decanoic acid	60,73,129,41	701	778	X	X	X
14.8	629-59-4	Tetradecane	57,43,71,85	865	932	X	X	X
14.95		Unknown (aldehyde structure)	41,57,82	917	959	X	X	X
15.2		Unknown (naphthalene structure)	156,141			X	X	
15.87		Unknown (alcohol structure)	55,41,69,83				X	X
16.28	4792-15-8 or 2615-15-8	Pentaethylene glycol or hexaethylene glycol	45, 89	700	794			X
16.38	56554-89-3	14-octadecenal	82,57,41	715	742	X	X	X
16.45	96-76-4	2,4-di-tert-butylphenol	191,206	884	906	X	X	X
16.8		Unknown (alcohol structure)	57,41,69,83			X	X	
16.9		Unknown (ester of carboxylic acid)	129, 111, 55, 83			X		
17.5	84-66-2	Diethyl phthalate	149,177	935	949	X	X	X
17.5		Unknown (alkane structure)	57,71,43,85	807	895	X	X	X
17.74	124-25-4	Tetradecanal	57,41,82	775	912	X	X	X
18.5		Unknown (alcohol structure)	43,55,69,83	915	936	X	X	X
18.7	24157-81-1	2,6-diisopropylnaphthalene	197,155,212	737	877	X	X	
18.8	629-78-7	Heptadecane	57,43,71,85	757	885	X	X	X
20.04		Unknown (alkane structure)	43, 57, 71, 85			X	X	X
20.18	118-60-5	2-ethylhexyl salicylate	120, 138, 250	738	890	X	X	
20.3	110-27-0	Isopropyl myristate	102, 228, 129, 185	720	758			X
20.8		Unknown (phthalate structure)	149, 223, 167			X	X	X
20.9		Unknown (alcohol structure)	69, 97, 55	789	847			X
21.46	82304-66-3	7,9-di-tert-butyl-1-oxaspiro[4.5]deca-6,9-diene-2,8-dione	205, 175, 189	843	858	X	X	X

SI: direct matching factor; RSI: reverse search matching factor.

**Table 4:** Tentatively identified polyester oligomers by LC-MS^n^.

TR (min)	*m/z* (Adduct) *	Product Ions	Proposed Compound	CT	Sample
16.6, 17.4	**584.3** (NH_4_^+^), 567.3 (H^+^), 589.3 (Na^+^), 605.3 (K^+^)		2PA+2CHDM (L)	I	CM3
18.8, 64.8	**419.2** (H^+^), 436.2 (NH_4_^+^), 441.2 (Na^+^), 457.2 (K^+^)	201, 149	PA+2CHDM	I	CM2, CM3
28.4	**636.3** (NH_4_^+^), 619.3 (H^+^), 641.3 (Na^+^), 657.3 (K^+^)		3PA+NPG+2EG (C)	III	CM2, CM3
29.5	**423.2** (Na^+^), 401.2 (H^+^), 418.3 (NH_4_^+^), 439.2 (K^+^)		2PA+NPG	I	CM2
35.5	**385.1** (H^+^), 407.1 (Na^+^), 423.0 (K^+^), 402.1 (NH_4_^+^)	193, 149, 341, 359	2PA+2EG (C)	III	CM2, CM3
39.7	**425.2** (Na^+^), 441.2 (K^+^), 420.2 (NH_4_^+^), 403.2 (H^+^)		2PA+2EG (L)	I	CM2, CM3
39.7	761.3 (H^+^), **778.4** (NH_4_^+^), 783.4 (Na^+^), 799.3 (K^+^)		3PA+2NPG+CHDM (L)	I	CM2, CM3
44.6	504.3 (NH_4_^+^), **509.3** (Na^+^), 525.2 (K^+^), 487.3 (H^+^)		2PA+2NPG (L)	I	CM2
51.6, 53.0	**683.4** (Na^+^), 678.3 (NH_4_^+^), 699.3 (K^+^)		3PA+3BD (C)	III	CM1
52.3	**427.1** (H^+^), 449.1 (Na^+^), 444.1 (NH_4_^+^), 465.1 (K^+^)	217, 149, 341, 193, 359	2PA+NPG+EG (C)	III	CM2, CM3
54.5, 55.6	**441.1** (H^+^), 463.1 (Na^+^), 458.1 (NH_4_^+^)	149, 167	2PA+CHDM	I	CM2
54.9, 56.3, 56.8	**469.2** (H^+^), 486.2 (NH_4_^+^), 507.1 (K^+^), 491.2 (Na^+^)	383, 235, 149, 162, 217, 401	2PA+2NPG (C)	III	CM1, CM2, CM3
58.6, 59.6	**483.2** (H^+^), 500.2 (NH_4_^+^), 505.2 (Na^+^), 521.1 (K^+^)	415, 231, 149, 383, 397	2PA+NPG+HD (C)	III	CM1
60.3, 60.7	**683.4** (Na^+^), 678.3 (NH_4_^+^), 699.3 (K^+^), 661.3 (H^+^)		3PA+2NPG+EG (C)	III	CM2, CM3
60.8, 61.9	**509.4** (H^+^), 531.4 (Na^+^)	491, 383, 235, 149, 257, 217	2PA+NPG+CHDM (C)	III	CM3
61.3	**497.2** (H^+^), 514.2 (NH_4_^+^), 519.2 (Na^+^), 535.1 (K^+^)	479, 415, 231, 149	2PA+2HD (C)	III	CM1
62.5, 62.8	725.3 (Na^+^), 720.3 (NH_4_^+^), **703.2** (H^+^), 741.2 (K^+^)	235, 469, 401	3PA+3NPG (C)	III	CM1, CM2, CM3
62.7	**781.5** (Na^+^), 797.4 (K^+^), 776.4 (NH_4_^+^)		3PA+2CHDM+EG (L)	I	CM2
63.4, 64.0	**917.2** (Na^+^), 912.2 (NH_4_^+^), 933.2 (K^+^), 895.2 (H^+^)		4PA+3NPG+EG (C)	III	CM3
63.6, 63.9	**739.3** (Na^+^), 734.3 (NH_4_^+^), 755.2 (K^+^), 717.3 (H^+^)		3PA+2NPG+HD (C)	III	CM1
64.4, 64.7	**753.3** (Na^+^), 748.3 (NH_4_^+^), 731.3 (H^+^), 769.2 (K^+^)		3PA+NPG+2HD (C)	III	CM1
65.2, 65.4	**767.3** (Na^+^), 762.3 (NH_4_^+^), 745.3 (H^+^), 783.2 (K^+^)		3PA+3HD (C)	III	CM1
63.9	**612.5** (NH_4_^+^), 617.5 (Na^+^), 633.4 (K^+^)		3PA+3EG (L)	I	CM2, CM3
64.8	743.3 (H^+^), 760.3 (NH_4_^+^), **765.3** (Na^+^), 781.2 (K^+^)		3PA+2NPG+CHDM (C)	III	CM3

* The adduct with the highest intensity is highlighted in bold text; CT: Cramer toxicity; L: linear; C: cyclic.

## Data Availability

The data presented in this study are available in [Characterization of Polyester Coatings Intended for Food Contact by Different Analytical Techniques and Migration Testing by LC-MS^n^].
